# Copy number profiles of paired primary and metastatic colorectal cancers

**DOI:** 10.18632/oncotarget.23277

**Published:** 2017-12-15

**Authors:** Futoshi Kawamata, Ann-Marie Patch, Katia Nones, Catherine Bond, Diane McKeone, Sally-Ann Pearson, Shigenori Homma, Cheng Liu, Lochlan Fennell, Troy Dumenil, Gunter Hartel, Nozomi Kobayasi, Hideki Yokoo, Moto Fukai, Hiroshi Nishihara, Toshiya Kamiyama, Matthew E. Burge, Christos S. Karapetis, Akinobu Taketomi, Barbara Leggett, Nicola Waddell, Vicki Whitehall

**Affiliations:** ^1^ Conjoint Gastroenterology Laboratory, QIMR Berghofer Medical Research Institute, Brisbane, Australia; ^2^ Hokkaido University Graduate School of Medicine, Sapporo, Japan; ^3^ Medical Genomics Laboratory, QIMR Berghofer Medical Research Institute, Brisbane, Australia; ^4^ Statistics Group, QIMR Berghofer Medical Research Institute, Brisbane, Australia; ^5^ The University of Queensland, Brisbane, Australia; ^6^ Royal Brisbane and Women’s Hospital, Brisbane, Australia; ^7^ Flinders University, Adelaide, Australia; ^8^ Pathology Queensland, Brisbane, Australia

**Keywords:** colorectal cancer, liver metastasis, loss of heterozygosity, copy number alterations, chemotherapy

## Abstract

Liver metastasis is the major cause of death following a diagnosis of colorectal cancer (CRC). In this study, we compared the copy number profiles of paired primary and liver metastatic CRC to better understand how the genomic structure of primary CRC differs from the metastasis. Paired primary and metastatic tumors from 16 patients and their adjacent normal tissue samples were analyzed using single nucleotide polymorphism arrays. Genome-wide chromosomal copy number alterations were assessed, with particular attention to 188 genes known to be somatically altered in CRC and 24 genes that are clinically actionable in CRC. These data were analyzed with respect to the timing of primary and metastatic tissue resection and with exposure to chemotherapy. The genomic differences between the tumor and paired metastases revealed an average copy number discordance of 22.0%. The pairs of tumor samples collected prior to treatment revealed significantly higher copy number differences compared to post-therapy liver metastases (*P* = 0.014). Loss of heterozygosity acquired in liver metastases was significantly higher in previously treated liver metastasis samples compared to treatment naive liver metastasis samples (*P* = 0.003). Amplification of the clinically actionable genes *ERBB2*, *FGFR1*, *PIK3CA* or *CDK8* was observed in the metastatic tissue of 4 patients but not in the paired primary CRC. These examples highlight the intra-patient genomic discrepancies that can occur between metastases and the primary tumors from which they arose. We propose that precision medicine strategies may therefore identify different actionable targets in metastatic tissue, compared to primary tumors, due to substantial genomic differences.

## INTRODUCTION

Colorectal cancer (CRC) is the third most common cancer worldwide, conferring significant morbidity, mortality and cost to the public health system [1]. The majority of deaths result from metastasis of the primary cancer to distant organs, predominantly the liver [2]. The standard first-line chemotherapeutic regimen for metastatic CRC is a combination of fluorouracil (5-FU) and folinic acid with oxaliplatin (FOLFOX) or irinotecan (FOLFIRI) with or without targeted agents [3]. Currently, the single molecular targetable treatment available for CRC is epidermal growth factor receptor (EGFR) inhibitors for tumors lacking mutation in the oncogene *KRAS* [4–6]. However, even among patients with *KRAS* wild type primary tumors, a recent study showed only 10–20 % of metastatic CRC patients benefitted from an EGFR inhibitor [7]. One contributory reason for this low rate of response may be discordance in *KRAS* mutation status between primary tumors and corresponding metastases [8, 9]. It is also likely for some cases that effector molecules downstream of the EGFR, including members of the MAPK and AKT signaling pathways [7], may be further altered during the metastatic process or following therapeutic intervention [10, 11]. Clarification of whether it is sufficient to assay the primary tumor or necessary to biopsy metastatic deposits for biomarker assessment is critical for optimizing management of patients with metastatic disease.

Genome-wide assessment of somatic copy number alterations provides opportunities for identifying cancer driver genes in an unbiased manner and may provide novel markers for the early diagnosis and personalized treatment of CRC [12]. For example, DNA copy number amplifications in *KRAS*, *ERBB2*, *MET* and *FGFR1* have been found to be predictors of poor prognosis and resistance to anti-EGFR therapy for CRC patients [13, 14]. It has also been recently shown that DNA copy number alterations are major sources of tumor heterogeneity, and one study found a striking discordance in copy number alterations between paired primary tumors and metastatic in samples from 27 patients using a DNA sequencing panel which was limited to only 100 genes [15]. Moreover, an understanding of the clonal composition of primary tumors and the extent to which this contributes to the molecular profile of metastatic disease pre- and post-therapy is vital to fully realize the potential of genomics in precision medicine approaches to improve patient outcomes.

We performed genome-wide chromosomal copy number assessment using single nucleotide polymorphism (SNP) arrays of primary CRC collected before therapy and the paired liver metastasis collected before or after therapeutic intervention. We show that copy number alterations differ significantly between primary tumors and their metastatic deposits.

## RESULTS

### Clinical and histologic data

**Figure 1:**
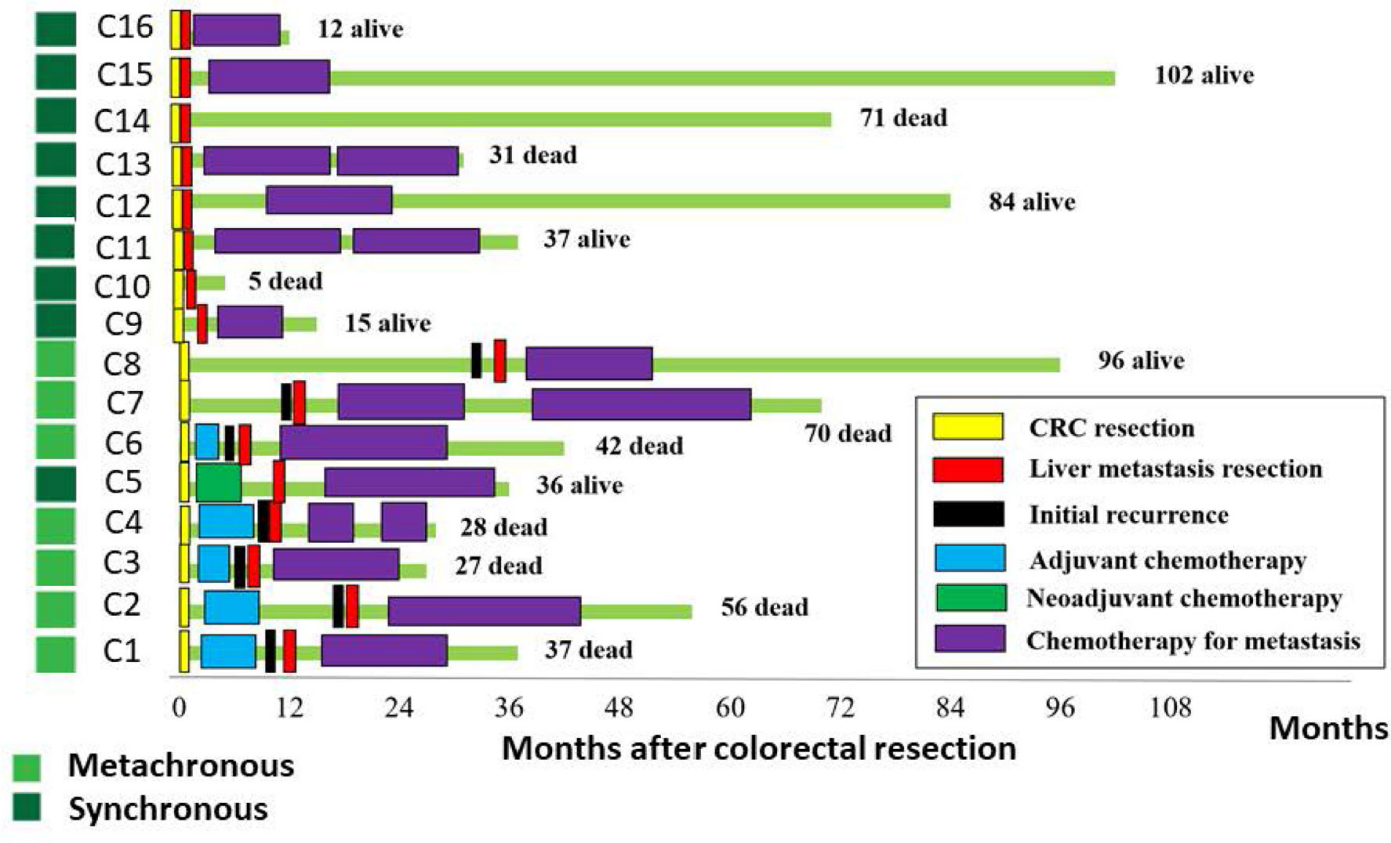
Clinical characteristics of the study cohort The course of treatment for the 16 CRC patients is indicated schematically. Samples were obtained at surgical resection time points indicated by yellow blocks for primary CRC and red blocks for the liver metastases. The survival status of the patient is shown, through observations made at 1–6 month intervals until death or December 2015.

Surgical samples of primary colorectal tumors and paired liver metastases from 16 patients (Median age 62.6 years) were analyzed (Table 1). A paired normal control sample was obtained for each patient from macroscopically normal mucosa adjacent to the primary tumor. In all 16 patients, primary CRC tissue was taken prior to chemotherapy. Nine patients had a synchronous liver metastasis at the time of diagnosis and 7 patients developed a liver metastasis metachronously (Figure 1). Metastatic tissue was taken after exposure to chemotherapy in 6 patients, which included 5 metachronous patients and 1 synchronous patient who had neoadjuvant chemotherapy prior to liver surgery (Figure 1).

**Table 1:** Clinicopathological characteristics of the 16 patients with CRC

Case No.	Age	Sex^*^	Location	CRC size (cm)	Invasion depth^^^	pN-factor	pStage at diagnosis	Adjuvant/Neoadjuvant chemotherapy ^#^	Chemotherapy for metastasis^#^	2nd chemotherapy^#^	Liver Metastasis Synchronous/ Metachronous with Primary	Outcome (months)
C1	67	F	Right side	3.6	SS	2	III	XELOX	XELOX		Metachronous	dead (37)
C2	57	F	Left side	4.0	SS	2	III	UFT+ LV	UFT+ LV		Metachronous	dead (56)
C3	74	M	Right side	5.5	SI	2	III	XELOX	XELOX		Metachronous	dead (27)
C4	63	F	Rectum	8.0	SE	3	^⋆^IV	mFOLFOX6	FOLFIRI		Metachronous	dead (28)
C5	64	M	Right side	7.0	SS	2	IV	XELOX+Bev	XELOX+Bev		Synchronous	alive (36)
C6	78	M	Rectum	6.0	SS	1	III	mFOLFOX6	FOLFIRI		Metachronous	dead (42)
C7	58	F	Rectum	3.0	SS	0	II	None	mFOLFOX6	FOLFIRI	Metachronous	dead (70)
C8	60	M	Left side	2.8	MP	0	II	None	TS1		Metachronous	alive (96)
C9	69	M	Rectum	4.6	SS	0	IV	None	mFOLFOX6+ Bev		Synchronous	alive (15)
C10	47	F	Right side	6.0	SE	3	IV	None	None		Synchronous	dead (5)
C11	69	M	Left side	6.0	SS	0	IV	None	XELOX+Bev	mFOLFOX6+ Bev	Synchronous	alive (37)
C12	52	F	Rectum	5.0	SS	3	IV	None	mFOLFOX6		Synchronous	alive (84)
C13	41	M	Rectum	5.0	A	2	IV	None	XELOX	IRIS+Bev	Synchronous	dead (31)
C14	73	M	Right side	7.0	SS	2	IV	None	None		Synchronous	dead (71)
C15	58	M	Left side	3.0	SS	1	IV	None	UFT+ LV		Synchronous	alive (102)
C16	72	M	Left side	4.8	SS	0	IV	None	mFOLFOX6		Synchronous	alive (12)

^*^M–male; F–female ^⋆^para aortic lymph node metastasis

^^^SS–Tumor invades through the muscularis propria into pericolorectal tissue; SI–tumor directly invades or is adherent to other organs or structures;

SE–tumor penetrates to the surface of the visceral peritoneum; MP–tumor invades muscularis propria; A–rectal tumor invades through the muscularis propria into pericolorectal tissue

^#^XELOX–Capecitabine and oxaliplatin; UFT+ LV–uracil-tegafur plus leucovorin; mFOLFOX6–fluorouracil (5-FU) and leucovorin with oxaliplatin;

FOLFIRI–5-FU and leucovorin with irinotecan; Bev–bevacizumab; TS1–Oral fluoropyrimidine anticancer drug; IRIS–irinotecan and TS-1.

*KRAS* mutational discordance between primary and paired metastatic tumors was identified in 2 patients (2/16, 13%), whilst *BRAF* mutation was concordant in the single patient with a *BRAF* mutant allele (Table 2). These two patients both had Stage III metachronous disease and had received adjuvant chemotherapy prior to sampling of the metastasis. Loss of MLH1 immunostaining, indicating a likely mismatch DNA repair deficiency and microsatellite instability, was detected in 3/16 patients (18.8 %, Table 2) and was concordant between all primary tumors and paired metastases (Supplementary Figure 1).

**Table 2:** Tumor purity and *KRAS*/*BRAF*/mismatch repair status in primary and paired metastasis

Case No.	Purity PT	Purity Met	*KRAS* PT	*KRAS* Met	*BRAF* PT	*BRAF* Met	Mismatch Repair PT	Mismatch Repair Met
C1	0.72	0.95	Mutant	Mutant	wt	wt	MSS	MSS
C2	0.87	0.88	wt	Mutant	wt	wt	MLH1 Loss	MLH1 Loss
C3	0.54	0.82	Mutant	Mutant	wt	wt	MSS	MSS
C4	0.63	0.78	wt	wt	wt	wt	MSS	MSS
C5	0.82	0.90	wt	wt	wt	wt	MSS	MSS
C6	0.64	0.92	Mutant	wt	wt	wt	MSS	MSS
C7	0.68	0.56	wt	wt	wt	wt	MLH1 Loss	MLH1 Loss
C8	0.70	0.98	wt	wt	wt	wt	MSS	MSS
C9	0.78	0.85	wt	wt	wt	wt	MSS	MSS
C10	0.90	0.75	wt	wt	Mutant	Mutant	MSS	MSS
C11	0.76	0.97	wt	wt	wt	wt	MSS	MSS
C12	0.92	0.96	wt	wt	wt	wt	MSS	MSS
C13	0.62	0.76	Mutant	Mutant	wt	wt	MLH1 Loss	MLH1 Loss
C14	0.90	0.88	Mutant	Mutant	wt	wt	MSS	MSS
C15	0.77	0.91	wt	wt	wt	wt	MSS	MSS
C16	0.73	0.79	Mutant	Mutant	wt	wt	MSS	MSS

PT: primary tumors Met: metastasis, wt: Wildtype, MSS: Microsatellite Stable.

Tumor purity was determined from SNP array data using qPure^38^.

### Differences between copy number and LOH profiles for each primary tumor and metastasis pair including the influence of chemotherapy exposure

**Figure 2:**
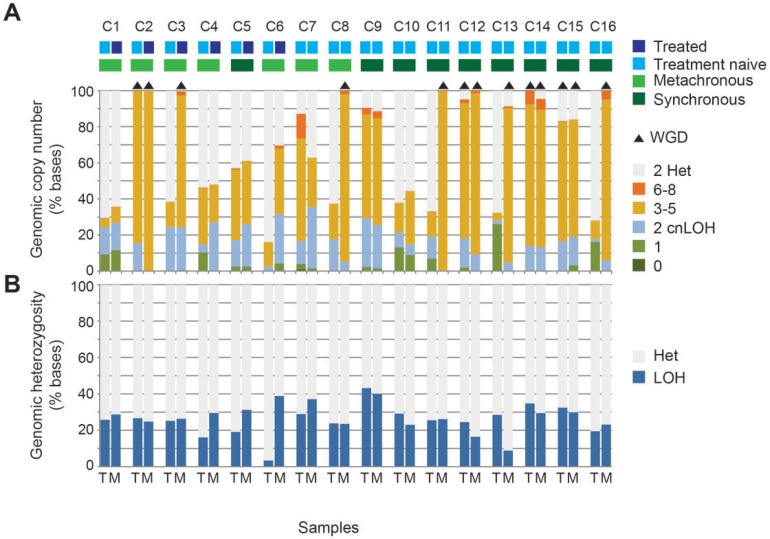
Differences between the copy number profiles of each tumor and metastasis pair including the influence of chemotherapy exposure The proportion of tumor genomes affected by copy number alteration and loss of heterozygosity (LOH) is shown for paired primary tumors (T) and metastases (M) from the 16 cases. The level of loss or gain of DNA is indicated by different colors (**A**). Whole genome duplication (WGD) is indicated by black triangles above the bar and is defined as >70% of at least half the chromosomes display copy numbers between 3 and 4 with both parental alleles present. The proportion of tumor genome with LOH is plotted (**B**).

We calculated the proportion of each genome that was affected by a copy number change from the normal diploid state. Within the 32 samples, the proportion of copy number altered genome ranged from 16.1% to 100%. In 16 samples, over 80% of the genome was copy number altered. Recent studies revealed that whole-genome duplication (WGD) events occur frequently during tumorigenesis and metastasis, resulting in cells bearing complex karyotypes [16, 17]. WGD was identified in both the primary tumor and metastasis in 4 pairs (C2, C12, C14 and C15) whereas in a further 5 pairs only the metastasis had undergone WGD (C3, C8, C11, C13 and C16. Figure 2A). There were no patients in whom only the primary tumor had undergone WGD. WGD was not significantly associated with synchronous versus metachronous disease state, or with treated versus treatment naive metastases.

Copy number alterations may result in shifts in the allelic ratio from the normal heterozygous state through to regions of loss of heterozygosity (LOH) that can occur at any copy number and is measured as the B allele frequency in the array data. For all sample pairs excluding from patient C6, the proportion of the tumor genome affected by LOH was similar between primary and metastatic tumors even when only the metastasis had undergone WGD (Figure 2B).

**Figure 3:**
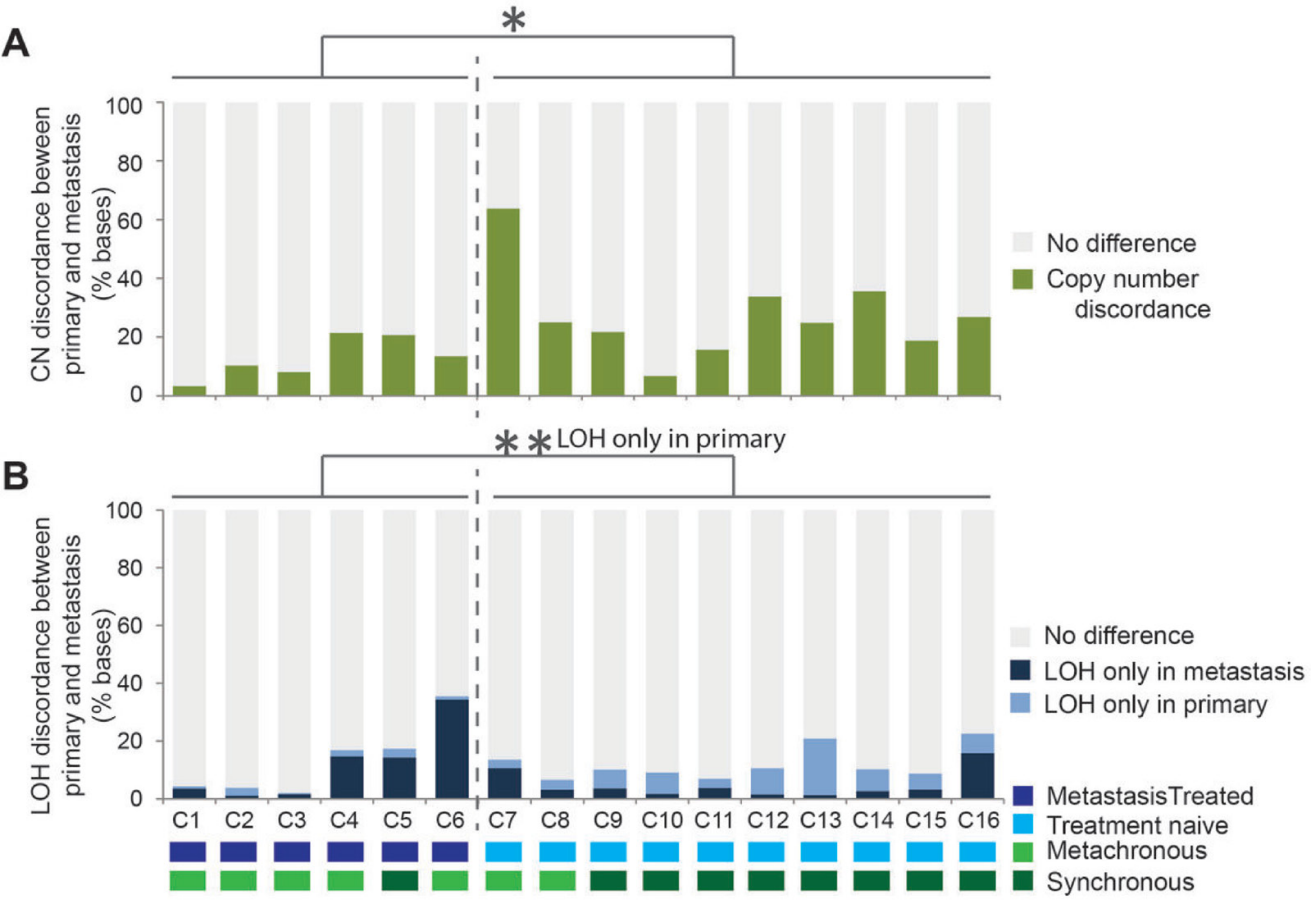
Genomic copy number and loss of heterozygosity (LOH) differences identified between all 16 primary tumor and metastasis pairs The copy number and LOH was assayed every 10Kb for 287921 sites per metastatic genome and compared with the corresponding region in the paired primary tumor sample with a correction for whole genome duplication applied to all sample pairs. The proportion of regions with copy number that was different between the paired samples is indicated in (**A**) by the height of the green bars. The proportion of regions for which LOH was observed only in the metastasis sample is indicated by the height of the dark blue bars and those with LOH only in the primary tumor sample by the height of the light blue sections (**B**). In both charts the grey indicates concordant copy number or LOH states between the paired samples. ^*^P < 0.05, ^**^P < 0.01 (*t*-test).

A comparative analysis was carried out to assess the differences in copy number states between the paired samples. Each genome was segmented into 10Kb regions and the copy number state was compared between corresponding regions in the paired primary and metastasis samples. Initially, in the 5 pairs of samples displaying WGD in only the metastasis, the genomes appeared highly different in metastases compared to matched primary tumors. On further examination, the majority of the differences were due an increase of 2 copies without a change in the LOH status. This large scale moderate copy number increase with no selection for one of the alleles is unlikely to result in driver gene expression changes relative to the background genes, therefore a correction for the WGD was applied. All regions where the copy number change difference was either +1 or +2 copies without LOH were recorded as no difference or concordant between paired samples. With the WGD correction applied, the average percent copy number discordance across all pairs of samples was 22.0% (range 3.3 – 63.6). Interestingly, the treatment naive tumor sample pairs had significantly higher copy number differences compared to sample pairs where the metastasis had been collected post-therapy (Figure 3A, *P* = 0.014). LOH events unique to primary samples occurred more frequently in those that had not been exposed to therapy, whilst LOH events unique to metastatic tumors was more frequent post-therapy (Figure 3B, *P* = 0.003). Overall the LOH patterns between the paired tumors were different by an average 12.4% of the genome (ranging from 2.0% to 35.6%, Figure 3B).

### Differences in candidate driver genes between primary and metastasis samples

**Figure 4:**
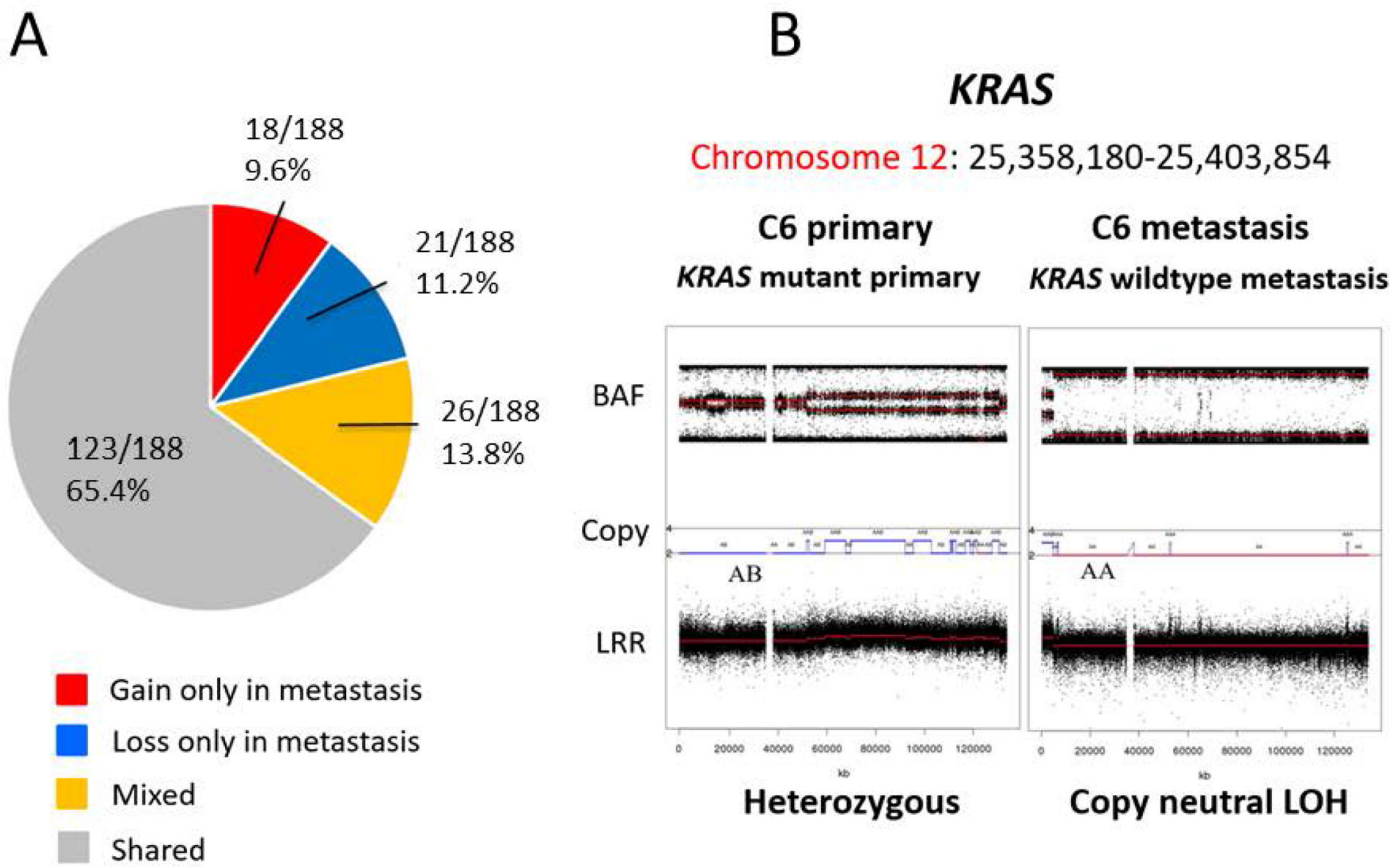
Differences in candidate driver genes between primary and metastasis samples We analysed the copy number status for 188 candidate CRC genes in the paired primary CRC and metastases (**A**). The copy number status of 123 genes (123/188 65.4%) was shared between primary and metastasis. *KRAS* mutation was present in the primary cancers but was not detectable in the metastatic sample following chemotherapy (**B**). The copy number profile of the genomic region containing *KRAS* indicates the locus was heterozygous (2 copies; AB) in the primary tumor but showed copy neutral LOH (2 copies; AA) in the paired metastasis (B).

We analyzed the copy number status for 188 candidate CRC genes in the paired primary tumors and metastases (Supplementary Table 1). The copy number status of 123 genes (123/188, 65.4%) was shared between primary tumor and metastasis (Figure 4A). These included genes recurrently mutated in colorectal cancer such as *APC*, *TP53*, *KRAS*, *EGFR, SMAD4, FBXW7*, *NRAS, VEGFA* and *RNF43*. The genes affected most frequently by discordant copy number status between primary and paired metastatic tissues were *CSMD3*, *TRPS1, TGFBR2*, *CTNNB1*, *FHIT* and *MACROD2* (Table 3)*. TGFBR2*, *CTNNB1* and *FHIT* are located on chromosome 3, which was amplified only in metastases (3/16, 18.8 %).

**Table 3:** Candidate gene list summarized to highlight differences in the copy number

Gene	Chromosome	Amplification private to metastasis	Loss private to metastasis
*CSMD3*	8	3/16 (18.8 %)	1/16 (6.3 %)
*TRPS1*	8	3/16 (18.8 %)	1/16 (6.3 %)
*TGFBR2*	3	3/16 (18.8 %)	0/16 (0 %)
*CTNNB1*	3	3/16 (18.8 %)	0/16 (0 %)
*FHIT*	3	3/16 (18.8 %)	0/16 (0 %)
*MACROD2*	20	0/16 (0 %)	3/16 (18.8 %)
*RBFOX1*	16	0/16 (0 %)	2/16 (12.5 %)
*PTK2*	8	2/16 (12.5 %)	2/16 (12.5 %)
*VPS13B*	8	2/16 (12.5 %)	1/16 (6.3 %)
*IRS2*	13	2/16 (12.5 %)	1/16 (6.3 %)
*CBLB*	3	2/16 (12.5 %)	0/16 (0 %)
*KALRN*	3	2/16 (12.5 %)	0/16 (0 %)
*WHSC1L1*	8	2/16 (12.5 %)	0/16 (0 %)
*FGFR1*	8	2/16 (12.5 %)	0/16 (0 %)
*MYC*	8	2/16 (12.5 %)	0/16 (0 %)

Colorectal cancer candidate genes are reported where copy number differences were observed between the paired samples for at least 2 cases.

An example of mutational discordance is shown in Figure 4B where *KRAS* mutation was detected in the primary tumor, which would have rendered the patient ineligible for anti-EGFR therapy. However, the paired liver metastasis was wild type for *KRAS*. The *KRAS* locus was heterozygous in the primary tumor but showed copy neutral LOH in the paired metastasis, suggesting deletion of the mutant allele in the metastatic tumor.

### Copy number status for genes with clinically available targeted agents

**Figure 5:**
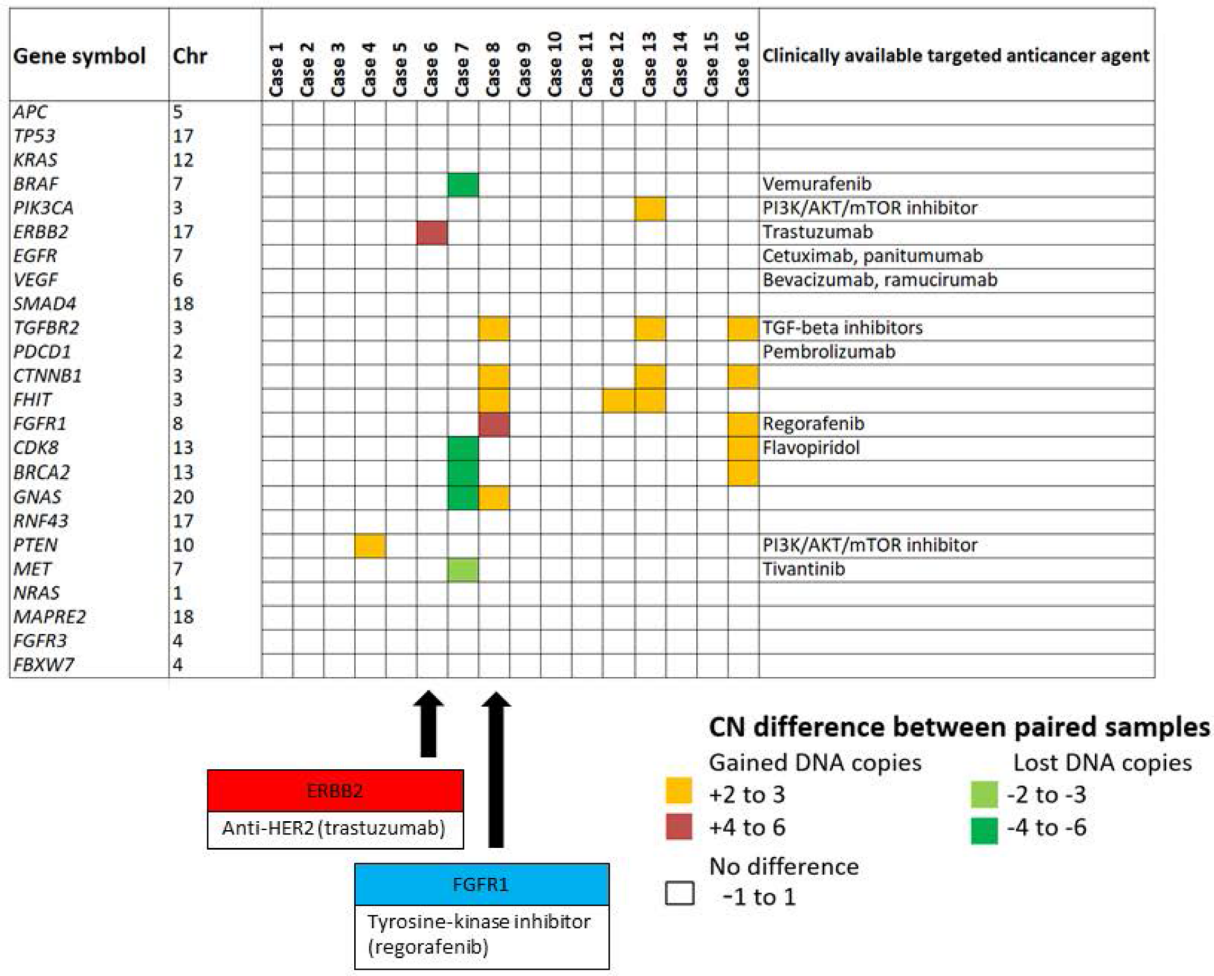
Copy number status private to the metastasis sample affecting clinically actionable targets The degree of copy number difference was calculated by subtraction of the primary tumor copy number state from that of the liver metastasis. The copy number status of 24 clinically actionable genes was assessed for alterations occurring specifically in metastatic but not primary tissue. *ERBB2*, a known target receptor of trastuzumab (anti-HER2), was specifically amplified only in the metastatic tissue of C6. Similarly, *FGFR1*, a known target receptor of regorafenib was specifically amplified only in the metastatic tissue of C8. Black arrow indicates specific gene amplification of C6 (*ERBB2*) and C8 (*FGFR1*) respectively.

The copy number status of 24 genes known to be clinically actionable in colorectal cancer [18, 19] were assessed for amplification occurring specifically in the metastasis but not the primary tumor, as these alterations may have been clinically useful in informing therapy selection. A subset of these can be targeted by therapies that are already approved or in late-phase development (Phase II or III). These include members of the RTK and RAS pathways, such as *KRAS*, *BRAF*, *NRAS*, and *ERBB2*, which have known associated with resistance to anti-EGFR targeted therapies [13]. In our study, amplification of *ERBB2, FGFR1, PIK3CA* or *CDK8* loci was observed in metastatic but not the paired primary tumors of patients C6, C8, C13 and C16, respectively (Figure 5). *ERBB2* encodes HER2, which is the target of trastuzumab. *ERBB2* was amplified only in the metastasis of patient C6, therefore trastuzumab may have been beneficial this patient following relapse from standard chemotherapy. Similarly, *FGFR1*, a target of regorafenib, was specifically amplified only in the metastasis of patient C8 (Figure 5).

## DISCUSSION

The significant morbidity of colorectal cancer is largely due to metastatic dissemination of the primary tumor. These distant metastatic deposits may have a very different molecular landscape to the primary disease as they may have arisen from minor clones of the primary disease or have undergone molecular changes through selective pressures during the metastatic process. However, actionable biomarkers used for informing therapeutic regimens for metastatic disease are usually based on the molecular status of the primary tumor sample alone. Here, we performed SNP arrays to investigate copy number profiles between paired primary and metastatic colorectal cancer samples to better understand the global differences that may occur between these two disease states.

When comparing the primary tumors to paired metastatic samples, almost a quarter (22.0%) of the genomes contained copy number differences. Whole genome duplication occurred in 4 primary tumors and their paired metastases. In another 5 paired samples, WGD was identified in the metastasis but not in the primary tumor. There were no paired samples where only the primary tumor had undergone WGD. A recent report revealed that the majority (62.5%) of esophageal adenocarcinoma undergo WGD and that tumors with WGD had different patterns of genomic alterations with more frequent oncogenic amplifications and less frequent inactivation of tumor suppressors [20]. Dewhurst *et al.* showed that in colorectal cancer, WGD allows for a higher rate of chromosomal instability, resulting in poor relapse-free survival [21]. These studies are consistent with our finding of greater rates of WGD in metastases compared to primary tumours. Further studies are now required to determine whether WGD is associated with distinctive clinical or molecular features.

In colorectal cancer patients with liver metastases, it has recently been reported that intra-tumoral heterogeneity is more common in patients previously exposed to chemotherapy compared with patients who are chemotherapy-naive [22]. A similar finding is seen in patients with urothelial carcinoma metastases [23]. Understanding how selective pressure from chemotherapy is involved in evolution from primary tumor to metastasis is a central biological question with clinical implications. In our study, LOH events unique to the primary tumor were commonly observed in therapy naive cases, which likely reflects the reduced clonal complexity of the secondary tumor due to the metastasis and expansion of a single primary tumor clone. By contrast, we predominantly observed an acquisition of LOH events in metastases following chemotherapy, suggesting the therapy has driven the increase in genomic complexity.

For colorectal cancer, the mutation status of *KRAS* and *BRAF* in is critical for directing choice of therapy. Here we have demonstrated a case where the primary tumor was *KRAS* mutant but was wildtype in the metastatic sample following chemotherapy. This suggests that the mutant allele was either lost from the metastatic tumor or the metastatic deposit arose from a KRAS wildtype clone in the primary tumor and highlights the importance of assessing clinically actionable markers in the metastatic rather than primary tumor sample. In this case, targeting the metastatic disease with an anti-EGFR agent may have improved the outcome for this patient.

Our data provide a strong rationale for investigating copy number variations throughout the genome to identify the full complement of potentially actionable genomic alterations that could be developed as clinical biomarkers or therapeutic targets. Amplifications may give rise to overexpression of oncoproteins that are targets of currently available drugs. We hypothesized that genes in regions of altered copy number would include known targets for currently available drugs that may be of benefit in the management of individual patients. We did find a number of examples including amplification private to the metastasis at loci for genes including *ERBB2*, *FGFR1, CDK8* and *PIK3CA*, which may have increased sensitivity to trastuzumab [24], regorafenib [25], flavopiridol [26], or PI3K/AKT/mTOR inhibitor, respectively (Figure 5). These metastasis-specific gene amplifications are at present insufficient to guide patient management. However, this demonstrates there are potentially actionable changes that occur exclusively in the metastatic tumor but not in the primary tumor.

Studies in lung, gastric, urothelial, thyroid and breast cancer have demonstrated somatic copy number discordance between primary tumors and their metastases [27–31]. Consistent with our results, these studies reported a high rate of concordance when examining known driver mutations or copy number alterations. In CRC, a recent report from Mamlouk is in line with these findings, however, they additionally validated cases of discordance in *MMP17*, *TCF7L2*, *GNAS*, *CARD11* and *TP53* at the level of gene copy number [15].

Here we provide proof of concept that metastatic samples can be substantially different from their matched primary tumors, even prior to chemotherapeutic intervention. Understanding intra-tumoral heterogeneity is clinically important because it could underlie failure of targeted systemic therapy. Further studies using more comprehensive approaches such as whole-genome sequencing will be needed to characterize all relevant classes of mutations, including single nucleotide variations, indels, copy number and structural rearrangements and non-protein coding mutations with regulatory significance between paired primary and metastatic cancers.

In conclusion, our results demonstrate that the molecular discordance between primary and metastatic tissue may be of clinical relevance in the era of genomically directed precision colorectal cancer medicine. Our data suggest that clinically actionable molecular targets for metastatic chemotherapy may be missed when relying only on biopsies of the primary tumor at the time of diagnosis. These differences could be driven by clonal evolution of the primary tumor due to the metastatic process or as a result of therapy. Serial metastatic or liquid biopsies obtained during the course of clinical care may improve outcomes by more accurately capturing the rapidly changing molecular landscape of a given patient’s disease to better rationalize strategies for personalized therapy.

## MATERIALS AND METHODS

### Human tissue samples

Samples from 16 patients who had undergone radical surgery for colorectal carcinoma between 2003 and 2014 at Hokkaido University Hospital were analyzed in this study. A total of 48 tissue samples from these 16 patients were obtained in a fresh state including from normal colorectal mucosa, primary tumors and the associated colorectal liver metastases. All tumors were snap frozen in liquid nitrogen within 20 minutes of extirpation. Normal tissue was sampled at least 10cm from the tumor margin. Clinicopathological information for the study patients is summarized in Table 1. Clinical staging and grading of liver metastases were based on the definition set by the Japanese Society for Cancer of Colon and Rectum Guidelines [32]. Patients were observed at 1–6 month intervals until death or December 2015. All patients provided written, informed consent. Approval was obtained from the Hokkaido University Human Research Ethics Committee (HREC) (14-005) and the QIMR Berghofer HREC (P2139). Nucleic acids were extracted using the Qiagen Allprep Kit in accordance with the manufacturer’s instruction. DNA was quantified using Qubit HS DNA Assay (Invitrogen).

### Clinical gene hotspot mutation testing

The presence of the *BRAF* V600E and *KRAS* codon 12 and 13 mutations were detected using allelic discrimination or high resolution melta analysis, respectively, as described previously [33–35]. Putative mutations were confirmed by Sanger sequencing.

### Immunohistochemical evaluation

Formalin-fixed paraffin-embedded tissue blocks were prepared from surgical specimens, and sections were stained with hematoxylin and eosin for histopathological examination by two gastrointestinal pathologists. Immunohistochemical staining for MLH1 was performed as described previously [36]. In brief, 4µM sections were cut, dewaxed and rehydrated. High pH antigen retrieval solution (pH 9.0; Dako, Glostrup, Denmark) was used for MLH1 at 112°C for 7 min. All sections were stained using the MLH1 antibody (clone G168-15, 1:100; BD Pharmingen). Loss of MLH1 expression was recorded when nuclear staining was observed in normal tissue but not in adjacent malignant cells.

### SNP arrays and copy number analysis

DNA from all samples was assayed with the Omni 2.5–8, V1.0 and V1.1 IlluminaBeadChips as per manufacturer’s instructions (Illumina). SNP arrays were scanned on an iScan (Illumina), data was processed using the Genotyping module (v.1.9.4) in GenomeStudio v.2011.1 (Illumina) to calculate B-allele frequencies (BAF) and logR ratios. GAP [37] software was used to segment the SNP array data and determine the level of copy number which was classified into one of 5 categories: homozygous deletion (copy number: 0), loss (copy number:1), copy neutral LOH (copy number:2), gain (copy number: 3–5) and amplification (copy number: 6–8). Loss of heterozygosity segments were generated using the segmented B-allele frequency data. Tumor cellularity was determined from SNP array data using qPure [38] to ensure a minimum tumor cellularity of 50%. Whole genome duplication (WGD) whereby >70% of at least half the chromosomes display copy numbers between 3 and 4 with both parental alleles present that typically totaled >45% of all genomic copy number (Supplementary Table 2). All array data has been deposited into the NCBI Gene Expression Omnibus (GEO) repository under accession GSE100787.

### Identification of discordant copy number and/or LOH between the sample pairs

A pair wise comparison analysis of genomic copy number and heterozygosity states over 10Kb windows for each primary and liver metastasis pair was carried out. The copy number and heterozygosity for each 10Kb was determined by the largest segment in that region identified using the GAP tool. Discordant copy number regions were identified by subtraction of the primary tumor copy number from that of the liver metastasis. To account for WGD inflating the number of discordant regions, all regions where the copy number change difference was either +1 or +2 copies without LOH were recorded as no difference or concordant between paired samples. Discordant regions of loss of heterozygosity were sought separately and classified into: unchanged; loss of heterozygosity in the liver metastasis; or retained heterozygosity in the liver metastasis. The genomic proportion of these discordant regions, that were made up of single or multiple adjacent 10Kb regions, was recorded.

### Genome-wide profiling of potential related genes

We sought to investigate the copy number state of 188 genes (listed in Supplementary Table 1) which were previously identified as significantly mutated genes in CRC [12, 15], and reported by The Cancer Genome Atlas (TGGA) [19] as well as the Catalogue of Somatic Mutations in Cancer (COSMIC) [39]. Copy number and LOH status for each gene was determined by the largest segment produced using GAP, within or spanning the gene footprint (from transcription start site to the last base of the longest transcript). Differences in copy number, heterozygosity status and segment boundaries between tumor pairs were summed across the patients.

### Statistical analysis

Two-tailed student’s *t*-tests were used to compare differences between groups. All differences were considered significant at a *p*-value of less than 0.05.

## SUPPLEMENTARY MATERIALS FIGURES AND TABLES







## Abbreviations

CRC: colorectal cancer
SNP: Single-Nucleotide-Polymorphism
LOH: loss of heterozygosity
MSI: microsatellite instability
MSS: microsatellite stable
BAF: B-allele frequency
WGD: whole-genome duplication
