# Supplementary material for: Copy number profiles of paired primary and metastatic colorectal cancers
Source: Oncotarget. 2017 Dec 15;9(3):3394–405. doi: 10.18632/oncotarget.23277 (PMC5790471; doi:10.18632/oncotarget.23277)
Supplement: Supplementary file 1 [file oncotarget-09-3394-s001.pdf]

Copy number profiles of paired primary and metastatic colorectal cancers

SUPPLEMENTARY MATERIALS

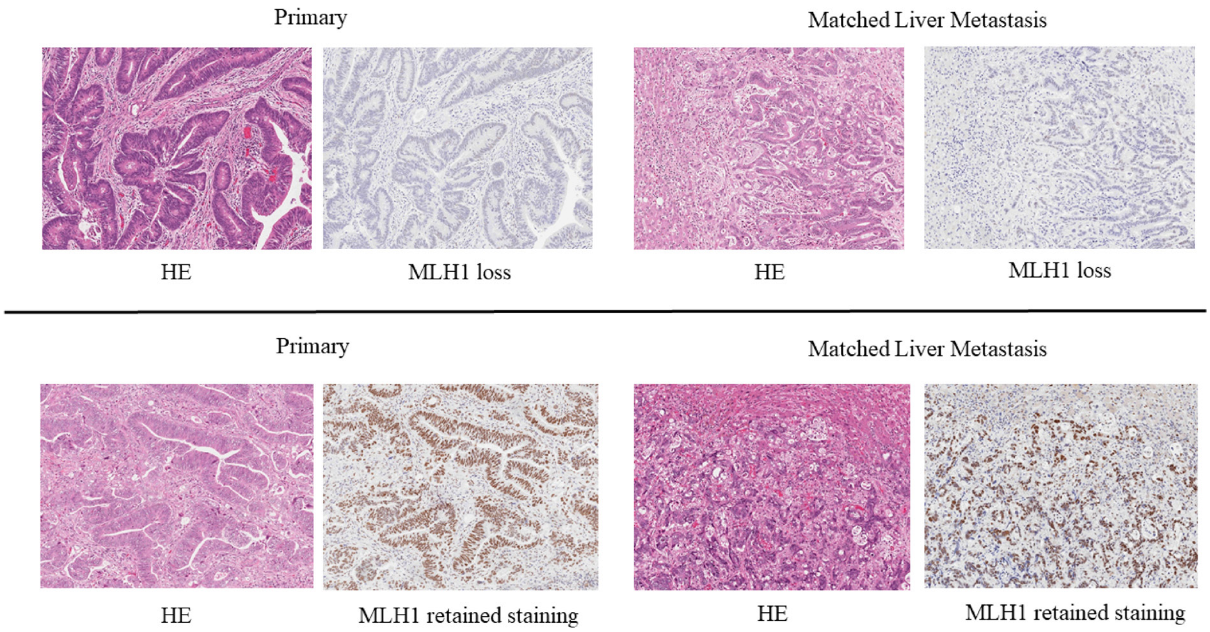

Supplementary Figure 1: MLH1 expression in primary and paired metastasis. Loss of MLH1 expression is shown in tumors from patient C13 (top half of figure) retention of MLH1 protein expression is shown in tumors from patient C9 (bottom half of figure).

Supplementary Table 1: The 188 colorectal cancer genes analyzed in this study. See Supplementary_Table_1

Supplementary Table 2: Identification of samples affected by whole genome duplication. See Supplementary_Table_2
